# Effect of negative dietary cation-anion difference (DCAD) diet on primiparous cow metabolomic analysis: Colostrum and milk assay

**DOI:** 10.5455/javar.2026.m1054

**Published:** 2026-06-22

**Authors:** Oscar David Aguilar-Vega, Héctor Aarón Lee-Rangel, Francisco Javier Castro-Lomelí, Ricardo Emmanuel Gaytan-Rodríguez, Anayeli Vázquez-Valladolid, Othoniel H. Aragón Martínez, Mario Alejandro Mejía-Delgadillo, Rogelio Flores Ramírez

**Affiliations:** 1 Facultad de Agronomía y Veterinaria, Centro de Biociencias, Universidad Autónoma de San Luis Potosí, San Luis Potosí 78321, México; 2 Facultad de Medicina, Universidad Autónoma de San Luis Potosí, San Luis Potosí 78210, México; 3 Facultad de Agronomía, Universidad Autónoma de Sinaloa, Culiacán, México; 4 Coordinación para la Innovación y Aplicación de la Ciencia y la Tecnología (CIACYT), Universidad Autónoma de San Luis Potosí, Av. Sierra Leona núm. 550-2ª, Lomas de San Luis, San Luis Potosí 78210, México

**Keywords:** Fatty acids, GC-MS, low negative anion-cation, metabolic disorders, prepartum

## Abstract

**Objectives:** This study aimed to characterize the metabolomic profile of colostrum and milk in primiparous Holstein cows subjected to a prepartum diet with a low negative dietary cation–anion difference (DCAD).

**Materials and Methods:** Eight primiparous cows were randomly assigned to two treatments: (a) basal diet (*n* = 4) and (b) basal diet supplemented with 250 gm/day of anionic salts (*n* = 4), starting 21 days before parturition. Colostrum and milk samples were analyzed using gas chromatography–mass spectrometry (GC–MS). Data were log-transformed and evaluated using multivariate (PLS-DA) and univariate (Wilcoxon test with fold change) approaches in MetaboAnalyst 6.0.

**Results:** A total of 34 and 31 metabolites were detected in colostrum and milk, respectively. Multivariate analysis showed a visual separation between treatments; however, model validation parameters indicated limited predictive performance. Volcano plot analysis did not identify metabolites with statistical significance (*p* < 0.05), although several compounds exhibited consistent trends in relative abundance between groups. In colostrum, fatty acids such as arachidic acid and cholesterol tended to be lower in DCAD-supplemented cows, whereas L-glutamate and dodecanedioic acid showed higher relative abundance. In milk, similar trend-level differences were observed in fatty acid profiles between treatments.

**Conclusions:** Prepartum supplementation with a negative DCAD diet was associated with exploratory metabolomic shifts in colostrum and milk of primiparous cows, particularly in lipid-related compounds. These findings should be interpreted as preliminary, and further studies with larger sample sizes are required to confirm the observed patterns.

## 1. Introduction

The transition period, encompassing the final three weeks of gestation through the first three weeks of lactation, is one of the most metabolically demanding phases in the productive cycle of dairy cattle. During this time, cows undergo extensive physiological, endocrine, and immunological changes to support fetal development, initiate lactation, and produce high-quality colostrum [1, 2]. Among the strategies employed to prevent transition-related diseases, prepartum feeding of diets with a low dietary cation-anion difference (DCAD) has proven effective in reducing the incidence of clinical and subclinical hypocalcemia. This approach enhances calcium mobilization by inducing a mild metabolic acidosis that sensitizes target tissues to parathyroid hormone and increases the efficiency of calcium absorption and resorption [3, 4]. While the effects of DCAD diets on mineral metabolism and health outcomes are well documented, less is known about their broader impact on systemic and mammary metabolism, particularly regarding the biochemical composition of colostrum and early lactation milk [5].

Primiparous cows constitute a distinct physiological group, as they must simultaneously allocate nutrients to complete their own growth while meeting the demands of gestation and the initiation of lactation. In comparison with multiparous cows, they generally exhibit reduced feed intake and slower endocrine adjustments, underscoring the importance of tailored nutritional strategies to ensure adequate support for maternal performance and fetal development [6, 7]. While the systemic metabolic responses to negative DCAD diets, such as shifts in plasma and urine metabolites, have been explored through metabolomics approaches, the extent to which these changes are reflected in the milk or colostrum metabolome remains unclear [8].

Colostrum is a highly specialized secretion critical for neonatal immunity and development. In addition to immunoglobulins and macronutrients, colostrum contains a complex mixture of bioactive compounds, including lipids, amino acids, and low-molecular-weight metabolites, many of which are sensitive to maternal nutrition and physiological state [9]. Milk composition during early lactation is also dynamically regulated and may reflect residual metabolic alterations from the prepartum period. The emergence of metabolomics has enabled a comprehensive analysis of small molecules in biological matrices, providing insights into the systemic effects of nutrition, disease, and physiological transitions. When applied to colostrum and milk, metabolomic profiling can reveal specific biomarkers of dietary interventions and identify functional compounds relevant to calf health and maternal adaptation [10].

We hypothesized that supplementation with anionic salts through a negative DCAD diet during the prepartum period would lead to measurable alterations in the metabolomic composition of colostrum and milk in primiparous cows. Therefore, the objective of this study was to characterize the metabolomic profile of colostrum and milk in primiparous dairy cows supplemented with a negative DCAD diet during the prepartum period.

## 2. Materials and Methods

### 2.1. Ethical approval

The study was conducted at a Holstein dairy unit of Autonomous University of San Luis Potosi, Soledad de Graciano Sanchez, San Luis Potosi, Mexico. The experimental procedures were submitted for review and approved by the Institutional Committee for the Care and Use of experimental animals of the Faculty of Agronomy and Veterinary (protocol ID: CICUAE/2024/03).

### 2.2. Animals and diets

Reproductive management included estrus synchronization and artificial insemination with sexed semen. Pregnancy diagnosis was performed by ultrasonography 35 days post-insemination, and non-pregnant cows were re-inseminated. Both insemination and pregnancy confirmation dates were recorded, and expected calving dates were calculated to ensure that dietary treatments commenced 21 days before parturition.

The experimental period lasted 51 days, consisting of 21 days prepartum for supplementation and 30 days postpartum for milk sampling. Eight primiparous Holstein cows (*n* = 4 per treatment; body condition score, BCS = 2.96 ± 0.11) were randomly assigned to one of two dietary treatments: (1) a control diet (Table 1), or (2) a diet supplemented with anionic salts to achieve a negative dietary cation–anion difference (DCAD). Cows in the treatment group received 250 gm/cow/day of a commercial anionic premix (PROMIN Reto Plus^®^, México), according to manufacturer recommendations. Diets were prepared daily and offered as a total mixed ration (TMR), with the anionic premix incorporated during the prepartum phase (21 days before calving). Urinary pH was measured once prior to parturition, averaging 6.6 for the control group and 5.5 for the negative DCAD group, confirming the expected acidogenic effect of the supplementation.

**Table 1.** Ingredients and chemical composition of dry prepartum cow diets.

Ingredient	kg of DM
Alfalfa	8.22
Corn Silage	3.425
Soybean meal	0.685
Corn grain flake	1.233
Mineral premix	0.137
Chemical composition	
DM, %	75.09
ME^1^	2.09
CP, %	13.78
aNDF^2^	49.74
EE, %	3.51
Ash, %	8.4
K^3^	5.26
S^3^	0.23
Na^3^	1.04
Cl^3^	1.34
DCAD^4^	127.96

^1^Metabolizable Energy; Mcal kg^–1^ DM^–1^) = Calculated according to NRC [32]. ^2^aNDF = amylase neutral detergent fiber organic matter. ^3^Values calculated according to Mendoza Martínez et al. [33]. ^4^DACD = (Na + K) – (Cl + S); mEq/kg of DM.

The cows were selected on the basis of their expected calving dates to ensure a homogeneous distribution of parturition time across treatments. Animals from both treatment groups were housed in group pens, with four cows allocated to each 300 m^2^ pen. The experimental facility consisted of a fan-shaped corral, designed to facilitate animal movement and management during the dry period. Each pen was equipped with shade structures for heat abatement, *ad libitum* access to fresh water, and daily refreshed bedding, which was mechanically tilled using a tractor to improve cow comfort and hygiene. These pens also served as the designated calving area, where parturition and early cow–calf management took place. Calves were separated from their dams within six hours after birth, following standard husbandry practices.

### 2.3. Samples collection and analytical methods

Immediately following parturition, individual cows were milked to collect colostrum, which was transferred into sanitized containers. The colostrum was gently homogenized using a whisk, and an aliquot was stored at –20 °C for subsequent analysis. Additionally, milk samples were obtained weekly from each cow until day 30 post-partum, consisting of 10 ml collected during three time points: an initial, an intermediate, and a final milking. These were pooled to create a composite sample of milk obtained weekly. All samples were frozen at –20 °C until analysis.

Colostrum and milk compounds analysis was conducted using a solvent mixture composed of 75% hexane and 25% acetone, following Perez-Segura et al. [11] protocol. Compound identification in colostrum and milk profiling were performed using gas chromatography (GC; HP 6890) coupled with mass spectrometry (MS; HP 5973), fitted with an HP 5MS capillary column (60 m × 0.255 mm × 0.25 µm film thickness; Agilent Technologies, CA, USA). The GC temperature program started at 70 °C (held for 2 min), followed by successive increases: to 250 °C at 20 °C/min, to 290 °C at 5 °C/min, then to 300 °C at 1 °C/min, and finally to 310 °C at 5 °C/min, where it was maintained for 36 min. The injector was set at 250 °C in splitless mode, with helium as the carrier gas flowing at 1 ml/min. Mass spectrometric detection operated in SCAN mode across a mass range of 50–500 m/z to identify compounds, following standard protocols for volatile compound profiling.

### 2.4. Metabolite identification and data processing

Compound identification was performed by comparing mass spectra with those available in the NIST mass spectral library, considering a similarity index ≥ 80% as acceptable for annotation. Only metabolites consistently detected in at least 50% of the samples within each treatment were retained for further analysis to reduce noise and improve data robustness. Peak areas obtained from GC-MS were used as a proxy for relative metabolite abundance, consistent with an untargeted metabolomic approach. Data were subsequently log-transformed prior to multivariate and univariate analyses to minimize heteroscedasticity and improve comparability across samples.

### 2.5. Statistical analysis

All statistical analyses were performed in MetaboAnalyst 6.0. GC-MS peak areas were used as relative abundance data in accordance with an untargeted metabolomic approach. Prior to analysis, data were log10-transformed to reduce heteroscedasticity and improve comparability among samples. Partial least squares discriminant analysis (PLS-DA) was applied as an exploratory multivariate tool to visualize overall clustering patterns between treatments and to identify metabolites contributing to group separation. Variable Importance in Projection (VIP) scores were extracted from the PLS-DA model and used only to rank the relative contribution of metabolites to the observed separation; VIP values were not interpreted as evidence of statistical significance. To assess model robustness, cross-validation parameters were examined, including leave-one-out cross-validation (LOOCV) accuracy, Q^2^, and permutation test results. Because of the limited sample size, these metrics were interpreted cautiously and used to support the exploratory nature of the multivariate analysis rather than to infer predictive performance. Univariate analysis was performed using volcano plots, combining log2 fold change and the Wilcoxon rank-sum test. Metabolites were considered statistically significant only when they met both criteria of *p* < 0.05 and absolute fold change > 1.0. Metabolites not meeting these thresholds were interpreted only as trend-level differences in relative abundance.

## 3. Results

### 3.1. Colostrum metabolomic analysis

**Figure 1.**
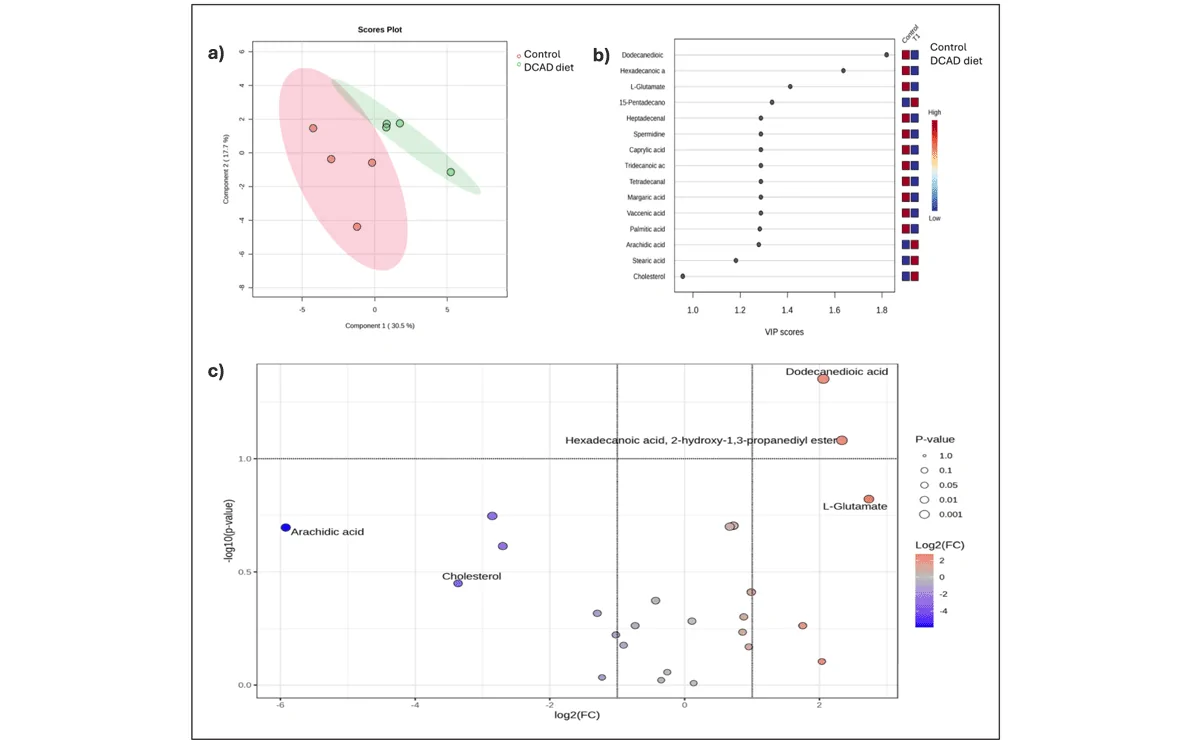
a) PLS-DA score plot derived from GC-MS metabolite profiling of colostrum samples. Each point corresponds to an individual sample, where red dots denote the control group, and green dots represent cows fed the negative DCAD diet. The x-axis and y-axis represent the variance explained by Principal Components 1 and 2, respectively; b) Loading plot showing the 15 most influential metabolites contributing to group separation between Control and negative DCAD-treated cows in colostrum samples; c) Volcano plot of the metabolomic analysis of the compounds detected and analyzed in first-calving bovine colostrum. The red circles represent the compounds that showed an overexpression in the control group, while the blue circles showed an increase in their activity in negative DCAD treatment.

A total of 34 metabolites were detected in colostrum samples from primiparous Holstein cows. The PLS-DA score plot showed a visual separation between control and DCAD-supplemented groups, with component 1 explaining 30.6% and component 2 explaining 17.7% of the total variance (Figure 1a). However, given the absence of robust model validation metrics, this separation should be interpreted as exploratory rather than predictive. Variable Importance in Projection (VIP) scores were used to identify metabolites contributing to group separation. Oleic acid, cis-vaccenic acid, and 2-hydroxypalmitic acid showed the highest VIP values (> 2.0), while eicosanoic acid, lactobacillic acid, and petroselinic acid presented moderate contributions (VIP >1.5) (Figure 1b). These metabolites contributed to the observed clustering pattern but should not be considered statistically discriminant.

The heatmap analysis suggested differences in relative abundance between groups, where oleic acid and cis-vaccenic acid tended to be greater in DCAD-supplemented cows, whereas lactobacillic acid and petroselinic acid were relatively higher in the control group. Volcano plot analysis did not identify metabolites meeting the predefined statistical significance threshold (*p* < 0.05). Nevertheless, some compounds, including cis-vaccenic acid, petroselinic acid, and lactobacillic acid, showed trend-level differences (log2 fold change > 1), indicating potential biological variation between treatments that warrants further investigation.

### 3.2. Milk metabolomic analysis

**Figure 2.**
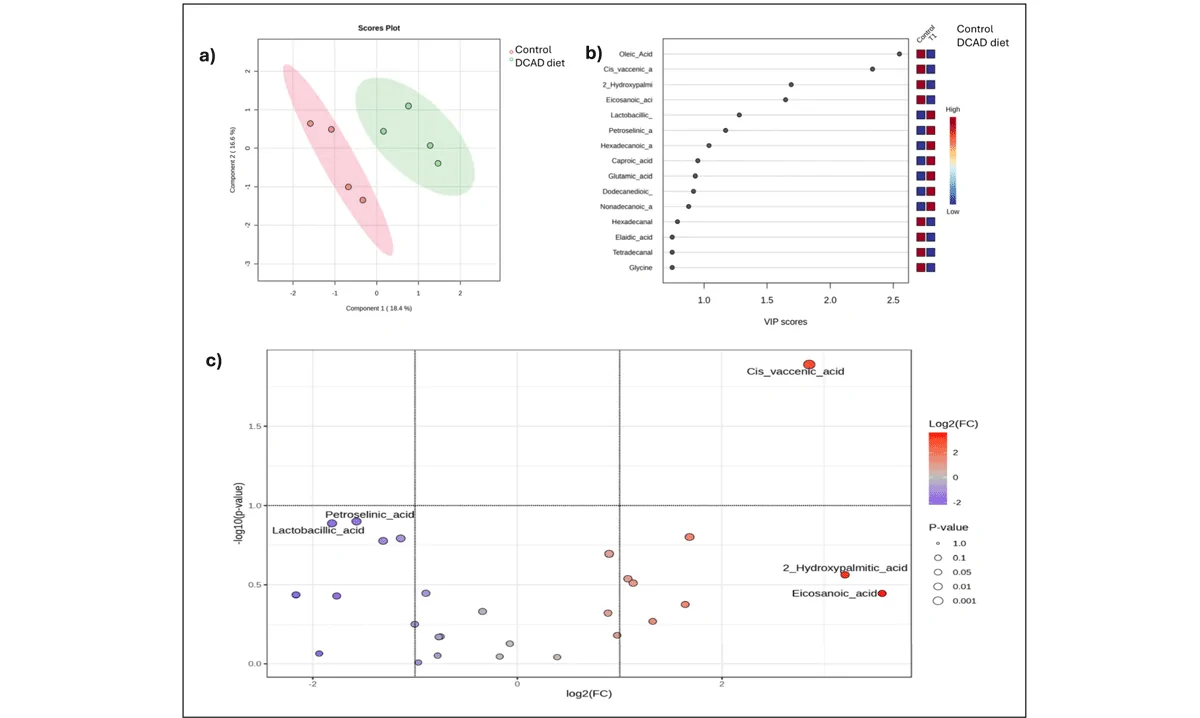
a) PLS-DA score plot derived from GC-MS metabolite profiling of milk samples. Each point corresponds to an individual sample, where red dots denote the control group, and green dots represent cows fed the negative DCAD diet. The x-axis and y-axis represent the variance explained by Principal Components 1 and 2, respectively; b) Loading plot showing the 15 most influential metabolites contributing to group separation between Control and negative DCAD-treated cows in milk samples; c) Volcano plot of the metabolomic analysis of the compounds detected and analyzed in first-calving bovine milk. The red circles represent the compounds that showed an overexpression in the control group, while the blue circles showed an increase in their activity in negative DCAD treatment.

A total of 31 metabolites were detected in milk samples. The PLS-DA score plot showed a visual separation between treatment groups along the first component (18.4% explained variance), with moderate clustering consistency (silhouette ≈ 0.53) (Figure 2a). However, cross-validation metrics indicated limited predictive capacity (Q²_LOOCV = –0.23; LOOCV accuracy = 0.38; permutation test *p* ≈ 0.31), suggesting that the observed separation should be interpreted with caution and considered exploratory. VIP scores ranged from 1.0 to 2.5 (Figure 2b). Oleic acid and cis-vaccenic acid exhibited the highest contributions (VIP ≥ 2.0), whereas glutamic acid, dodecanedioic acid, caproic acid, lactobacillic acid, and petroselinic acid showed values close to 1.0, indicating borderline contributions rather than robust discriminant features.

Heatmap visualization suggested differences in metabolite abundance patterns between treatments. Cis-vaccenic acid, glutamic acid, and dodecanedioic acid tended to be higher in the DCAD group, whereas lactobacillic acid, petroselinic acid, and caproic acid tended to be higher in the control group.

Volcano plot analysis revealed that none of the metabolites exceeded the statistical significance threshold (*p* < 0.05). Some compounds, such as arachidic acid and cholesterol, tended to be higher in the control group, whereas L-glutamate and dodecanedioic acid showed higher relative abundance in the DCAD group. These differences should be interpreted as trends rather than statistically significant changes.

## 4. Discussion

Bovine colostrum is essential for calf survival, health, and early development, providing immunoglobulins, growth factors, antimicrobial peptides, and metabolic regulators [12]. While research has traditionally focused on macronutrients and fatty acids, metabolomics now offers a more comprehensive perspective of the molecular changes occurring during the transition from colostrum to mature milk. Metabolomic approaches have frequently used plasma as a reference matrix due to its systemic coverage. However, milk has gained relevance as a non-invasive and practical alternative, as it directly reflects mammary metabolism and can be easily collected during routine dairy operations [13, 14]. In this study, the metabolomic profile of colostrum and milk included compounds previously associated with sensory and flavor attributes in dairy products. This information may help explain potential differences in consumer perception, particularly regarding the presence or absence of off-flavors [15]. Untargeted metabolomics typically yields a broad chemical fingerprint, including alcohols, hydrocarbons, aromatic compounds, ketones, aldehydes, and fatty acids; however, their concentrations may vary depending on storage time and conditions [11, 16].

Metabolomic analysis of colostrum from primiparous cows supplemented with anionic salts revealed variations in specific bioactive metabolites, suggesting that DCAD supplementation may influence mammary metabolism and, consequently, colostrum composition through changes in systemic acid–base balance and mineral homeostasis [17]. Colostrum from cows supplemented with a negative DCAD diet tended to show higher relative abundance of arachidic acid (C20:0), stearic acid (C18:0), and cholesterol. These long-chain fatty acids and lipids are important for membrane composition, energy metabolism, and neonatal development [18, 19, 20]. Their relative abundance may suggest potential alterations in lipid transport and mobilization associated with acid–base balance and mineral metabolism during the prepartum period [21, 22], with possible implications for immunity transfer and calf development [23, 24]. Conversely, colostrum from control cows tended to show higher relative abundance of L-glutamate, palmitic acid (C16:0), and dodecanedioic acid (C12:0). Glutamate, a multifunctional amino acid, participates in energy metabolism, nitrogen balance, and oxidative stress regulation [25, 26, 27]. Its relative abundance in control animals may reflect differences in amino acid metabolism not observed under DCAD supplementation.

In milk, supplementation with a negative DCAD diet was associated with variations in the relative abundance of several fatty acids. Lactobacillic acid, petroselinic acid, and caproic acid tended to be higher in control cows, whereas cis-vaccenic acid, glutamic acid, and dodecanedioic acid tended to be higher in the DCAD group. Lactobacillic acid, a microbial-derived fatty acid, tended to decrease under DCAD supplementation, which may suggest a reduced microbial contribution to the milk metabolome [28, 29]. Similarly, petroselinic acid, an isomer of oleic acid with reported anti-inflammatory properties, tended to be lower in the DCAD group, consistent with potential shifts in lipid metabolism [30, 31]. In contrast, cis-vaccenic acid tended to increase in the DCAD group, which may be associated with stimulation of de novo fatty acid synthesis and possible changes in ruminal fermentation patterns. The presence of hexadecanoic, octadecanoic, and 9z-octadecanoic acids in milk fat has been previously associated with metabolic imbalances such as subclinical ketosis. These compounds have been reported in hyperketonemic ewes, suggesting that elevated concentrations of these fatty acids may serve as potential indicators of metabolic disturbances [11].

Despite the controlled experimental conditions and the use of primiparous cows to reduce biological variability, the relatively small sample size (*n* = 4 per treatment) represents an important limitation of this study. This constraint may reduce the statistical power to detect significant differences and limits the generalizability of the findings. Therefore, the results should be interpreted within an exploratory metabolomic framework, where the objective is to identify potential patterns and generate biological hypotheses rather than to establish definitive causal relationships. It is important to note that untargeted metabolomic approaches are highly sensitive and can reveal subtle metabolic shifts even in studies with limited sample size; however, these observations require validation in larger and independent cohorts. Future research should include increased numbers of animals and targeted analytical approaches to confirm the trends identified in this study and to strengthen their biological relevance.

## 5. Conclusions

The prepartum supplementation of a negative DCAD diet was associated with exploratory changes in the metabolomic profile of colostrum and milk in primiparous cows, particularly in lipid-related compounds. These findings provide preliminary insights into metabolic adaptations induced by DCAD strategies; however, they should be interpreted with caution due to the limited sample size. Further studies with larger populations are required to confirm these observations.

## Data Availability

The data presented in this study are available from the corresponding author upon reasonable request.

## List of abbreviations

DCAD, dietary cation–anion difference; GC-MS, gas chromaography masas coupled; CICUAE, committee for the care and use of experimental animals; BCS, body condition score; TMR, total mixed ration; PLS-DA, partial least squares discriminant analysis; VIP, Variable Importance in Projection.
